# Managing microbial keratitis in resource-limited settings

**Published:** 2025-01-31

**Authors:** Jeremy Hoffman, Reena Yadav, Abel Ebong, Simon Arunga, Astrid Leck

**Affiliations:** 1 Consultant Ophthalmologist and Corneal Service Lead: Buckinghamshire Healthcare NHS Trust and Clinical Research Fellow: International Centre for Eye Health, LSHTM, UK.; 2 Consultant Ophthalmologist and Head of Department, Cornea: Sagarmatha Choudhary Eye Hospital (SCEH), Lahan, Nepal.; 3 Ophthalmologist: Mbarara University of Science and Technology, Mbarara, Uganda.; 4 Senior Lecturer, Department of Ophthalmology: Mbarara University of Science and Technology and Honorary Assistant Professor: International Centre for Eye Health, LSHTM, UK.; 5 Assistant Professor: International Centre for Eye Health, LSHTM, London, UK.

**In resource-limited settings, the management of corneal infections (microbial keratitis) is challenging. Patients often delay seeking medical help, arriving in clinic with advanced disease, and diagnostic laboratory support may be unavailable.**

**Figure:**
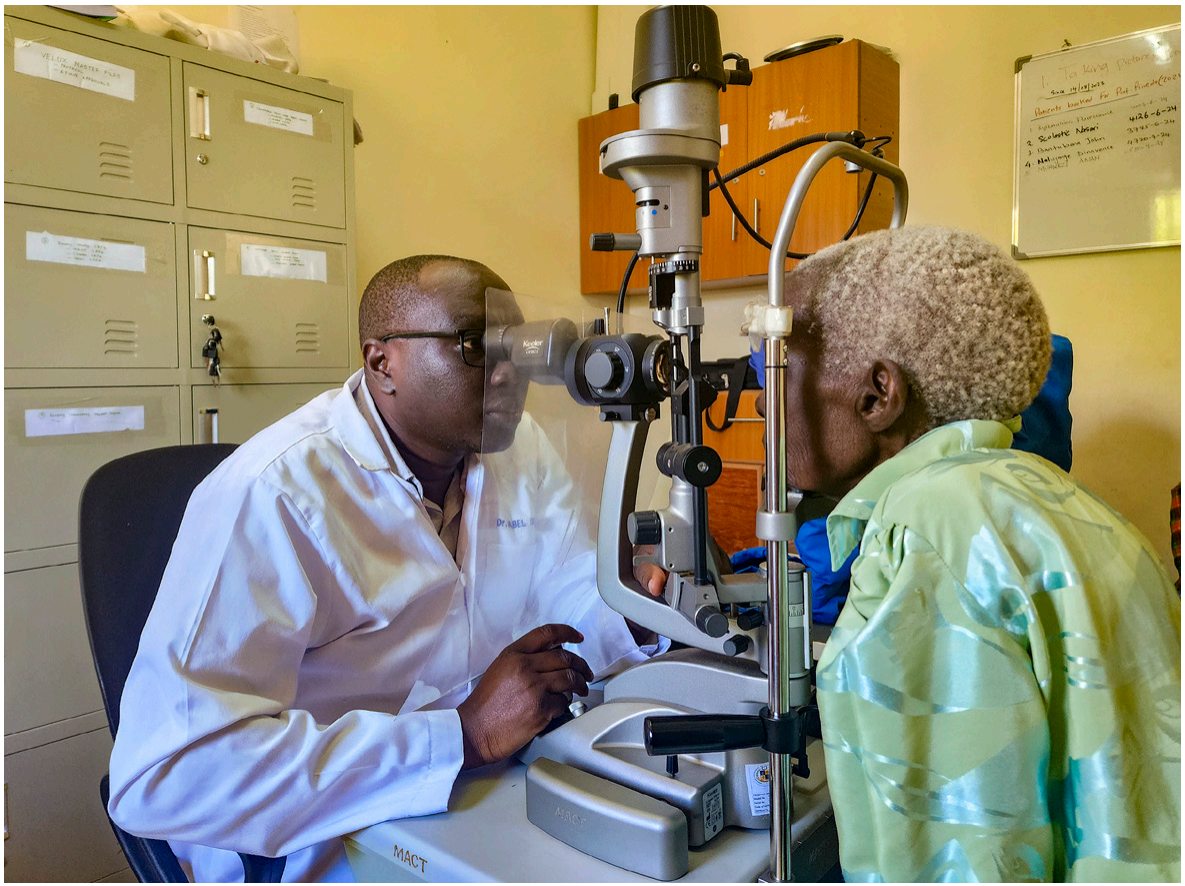
Examining a patient with suspected corneal infection. UGANDA

© ABEL EBONG CC BY-NC-SA 4.0

## **PART 1** General management principles

In this section, we discuss the general principles of managing a patient with microbial keratitis.

Patients with microbial keratitis need:

1. Early and appropriate diagnosis and treatment, ideally based on microbiology results
2. Good pain management
3. Good adherence with treatment during the first few days (usually achieved by admitting the patient)
4. Regular clinical review.

### Early diagnosis and treatment

If there are clinical signs or symptoms to suggest microbial keratitis or corneal infection (see poster on pages 8–9), start patients on a broad-spectrum topical antibiotic and refer them urgently to a specialist eye clinic.

Once reviewed in hospital, most patients can be effectively managed with intensive topical treatment alone, but there are some patients for whom supportive or adjunctive treatment may be needed.

Ideally, the treatment given should be based on microbiological identification of the microorganism involved. In the absence of microbiological services, we suggest you refer to the article on page 3 to guide your decision making. In settings where fungal keratitis is known to be more common than bacterial keratitis, first-line treatment should include antifungal eye drops. If there is any uncertainty about the pathogen responsible, it is advisable to treat empirically with both antifungal and antibiotic eye drops, until a definitive diagnosis can be made.

For most patients with microbial keratitis, treatment typically follows an intensive ‘sterilisation’ phase for up to a week, where drops are instilled every hour. This is followed by a ‘healing’ stage, where the intensity of the drops is reduced to allow the corneal epithelium to heal. The timings and antimicrobial eye drops vary depending on the causative microorganisms.

For more information on the different medical treatment needed for bacterial, fungal and *Acanthamoeba* keratitis, see Part 2.

### 2. Pain management

Microbial keratitis causes severe pain. It is therefore important to manage patients’ pain, as pain is often one of the reasons a patient may start using traditional eye medications or inappropriate conventional medicines, such as topical steroids. We recommend cycloplegia (with topical cyclopentolate or atropine), along with oral painkillers such as paracetamol, codeine, or NSAIDs such as ibuprofen.

### 3. Adherence and counselling

Management of microbial keratitis requires a multi-disciplinary approach. It is vital to counsel the patient about the disease, its management, the expected outcome, and the need for long-term follow-up.

Many patients do not get adequate information about how to continue their treatment after being discharged, such as how often to instil their eye drops and how to position themselves to ensure the drops go into the eye. This contributes to inadequate adherence with medication.

Long-term follow-up (of about 2–3 months) is often needed; if patients do not realise the importance of this from the outset, they frequently miss future appointments, which can lead to adverse clinical outcomes.

### 4. Monitoring and follow-up

Follow-up is essential to evaluate the effectiveness of prescribed medication in patients with microbial keratitis.

Follow-up frequency depends on disease severity; for most patients a review at 48–72 hours is recommended when initial symptomatic improvement (typically a reduction in pain and discharge) can be observed, along with reviewing any initial microbiology results. Patients with severe microbial keratitis may require admission (if available) and daily monitoring until stable, due to the risk of corneal perforation. If patients have to travel from far away, they should be admitted for as long as possible, ideally for the first few days of treatment at a minimum.

Signs that the patient is getting better include reduced pain, reduced redness and anterior chamber activity, and a reduction in the size of the epithelial defect. However, in fungal keratitis (and in certain forms of bacterial keratitis, such as *Pseudomonas* keratitis), there can be an initial worsening of clinical signs due to the inflammatory response to dead or dying pathogens. Drug toxicity may also delay the healing process, which requires close monitoring. A reduction in pain and eyelid swelling may be the only initial positive findings.

If patients are not improving, then there are several questions to consider. First, what is their treatment adherence like? Are they using the drops as prescribed? Second, do they have an underlying (often unknown) systemic condition that is responsible, such as uncontrolled diabetes or HIV? Third, is this a mixed bacterial and fungal infection? Finally, are they using traditional eye medicine that is making things worse?

## **PART 2** Medical treatment

Treatment can be broadly categorised into medical and surgical options. Medical management typically involves the use of topical antimicrobial medication. When medical management is insufficient, or when there are complications, surgical interventions such as corneal debridement, therapeutic keratoplasty, or conjunctival flaps may be needed to preserve vision and the structural integrity of the eye.

In this section, we discuss the medical management of bacterial, fungal, and *Acanthamoeba* microbial keratitis in a low-resource setting.

### A note about corticosteroids

The adjunctive use of topical corticosteroid therapy in microbial keratitis remains hotly debated. While corticosteroids may suppress inflammation and reduce scarring and associated visual loss, potential disadvantages include infection recurrence, local immunosuppression, corneal melting risk, and increased intraocular pressure. Patients already on corticosteroids should reduce or stop them until the infection is under control.

Topical corticosteroids may be considered for patients with severe bacterial keratitis with signs of significant acute inflammation, and if there is clinical improvement following at least 48 hours of intensive topical antibiotics. The dosing frequency should be the minimum amount to control the inflammation.

Steroids should **not** be prescribed for fungal, *Acanthamoeba* or *Nocardia* corneal infections.

### Other adjunctive therapy

Oral antibiotics are not usually required unless there is a corneal perforation, when an oral fluroquinolone can be used, e.g. moxifloxacin. There may be a role for oral doxycycline 100 mg once daily to help limit corneal melting and scar formation, particularly in patients with more severe disease.

For patients with deep fungal keratitis, or where there is failure to respond to initial natamycin 5% treatment, oral antifungals such as voriconazole or itraconazole may be given. However, these drugs have dangerous side effects including liver toxicity and therefore should only be given where liver function testing can be performed. Additional treatment for these more challenging, deeper fungal infections includes injections of amphotericin B or voriconazole into the anterior chamber (intracameral injections), or intrastromal around the infiltrate. However, there is little convincing evidence to support the use of intrastromal injections.

## Bacterial keratitis

It is always important to base antibiotic choice on local antimicrobial susceptibility profiles if such data are available.

Ideally, admit the patient initially to ensure that the eye drops are administered as planned and to allow regular follow-up. Give treatment as per the frequency in Table 1.

**Table 1:** Bacterial keratitis: treatment summary

**Indication**	**Drugs**	**Intensity and duration**
Initial or first-line treatment	Moxifloxacin, ofloxacin, **or** ciprofloxacin eye drops	Hourly drops, day and night, for 48 hours, then hourly during the day for 3–5 days, then four times a day until the epithelial defect has healed
Patients with central or severe keratitis	Cefuroxime **and** gentamicin eye drops	As above
Patients with deep or large ulcers	Oral doxycycline 100 mg once a day for deep/large ulcers. After 48 hours, consider adding chloramphenicol 1% ointment at bedtime	

**Single-drug therapy** with topical fluoroquinolones is as effective as combination therapy (see below). Fluoroquinolone options include ciprofloxacin, ofloxacin, levofloxacin, moxifloxacin and gatifloxacin.

**Combination therapy** – using a combination of topical cephalosporin (e.g., cefuroxime) and topical gentamicin – can be considered in patients with central or severe keratitis (generally defined as an infiltrate of greater than 2 mm or more), particularly in patients with a hypopyon, or in patients who are unresponsive to initial single-drug therapy with fluoroquinolone.

Ointments are less effective, due to poor corneal penetration, but may be used adjunctively or at bedtime in patients with milder infection. Subconjunctival injections may be considered for patients with adherence difficulties or if there are delays in obtaining fortified antibiotic eye drops. Systemic therapy is reserved for patients with scleritis, endophthalmitis, microbial keratitis associated with systemic infections, or gonococcal keratitis.

**Note:** In patients with bacterial keratitis (particularly if caused by *Pseudomonas* spp.), the clinical signs can often be worse in the first few days after starting treatment; for example, the size of the hypopyon may increase. However, if the patient's pain is reducing significantly, this is a reassuring sign that the treatment is working. Treatment should then continue unchanged, and the patient be reviewed further in a few days’ time.

## Fungal keratitis

The standard treatment for fungal keratitis is natamycin 5% drops. The treatment schedule is given in Table 2.

**Table 2:** Fungal keratitis: treatment summary

**Indication**	**Drug**	**Intensity and duration**
Initial or first-line treatment for filamentous fungal keratitis	Natamycin 5% eye drops	Hourly drops day and night for 48 hours, then hourly during the day for 5 days, then 2-hourly for 7 days, reducing to 4–6 times per day once there are signs of epithelium healing, followed by 4 times per day until the epithelium is fully healed
If natamycin is not available	Chlorhexidine 0.2% **or** voriconazole 1% eye drops	As above
First-line treatment for non-filamentous keratitis (e.g. yeast or *Candida* spp.). Adjunctive treatment for recalcitrant filamentous fungal keratitis	Amphotericin B 0.15% eye drops	As above
To prevent secondary bacterial infection while there is an epithelial defect	Quinolone antibiotic (such as moxifloxacin, ciprofloxacin, or ofloxacin eye drops)	Administer 4 times a day until the epithelium or ulcer has healed

Voriconazole 0.1% drops may be considered if natamycin is not available, although it may be prohibitively expensive. In the absence of commercially produced antifungal eye drops, 0.2% chlorhexidine drops can be considered an alternative treatment. Chlorhexidine has the advantage of being inexpensive and easy to formulate (see article in issue #118 www.cehjournal.org/articles/285).

Amphotericin B 0.15% eye drops have also been used in recalcitrant cases of filamentous fungal keratitis, and remains first-line treatment for patients with non-filamentous *Candida* keratitis. This could be made in a hospital, as per the instructions in the panel.

**Amphotericin B 0.15% eye drop preparation**

**What you need:**

- A 50 mg vial of liposomal amphotericin B parenteral powder for injection (the type used to constitute an IV drip; brands from India include Amfocare)
- Distilled, sterilised water
- Artificial tears (eye drops)
- Sterile eye drop bottle (minimum 10 ml).

**Method**

- Mix the 50 mg vial of liposomal amphotericin B parenteral powder with 10 ml of distilled, sterilised water. It should fully dissolve without any precipitates.
- Add 3 ml of this preparation to the eye drop bottle, followed by 7 ml of artificial tears (eye drops).
- Store the solution at 4 degrees Celsius; it can be used for 1 week.

While there is an epithelial defect, use a topical broad-spectrum quinolone antibiotic (such as moxifloxacin, ciprofloxacin, or ofloxacin, depending on local availability and resistance patterns) to prevent a secondary bacterial infection. This is administered four-times per day until the epithelium heals.

As with bacterial infections, the patient should be admitted initially to ensure the eye drops are administered as planned and to allow regular follow-up.

## *Acanthamoeba* keratitis

*Acanthamoeba* keratitis poses a challenge in treatment due to its resistant cyst stage, which is less responsive to therapeutic agents compared to the trophozoite stage. Biguanides, particularly chlorhexidine and polyhexamethylene biguanide (PHMB), are commonly used drugs, either alone or in combination with diamidines.

PHMB 0.06-0.08% monotherapy can be used as first-line treatment for *Acanthamoeba* keratitis. However, in resource-limited settings where fungal keratitis is also prevalent and PHMB may not be available, it is pragmatic to use chlorhexidine (typically 0.02%, but 0.2% can be used if that is all that is available), ideally in combination with a diamidine such as propamidine isethionate 0.1% and hexamidine 0.1%. However, prolonged use of propamidine isethionate may lead to toxic keratopathy, as well as iris atrophy, cataract, and peripheral ulcerative keratitis.^1^

Initial therapy for *Acanthamoeba* keratitis involves frequent administration (Table 3), gradually tapering based on response, with treatment durations ranging from 3 months to over a year.

**Table 3:** *Acanthamoeba* keratitis: treatment summary

**Indication**	**Drug**	**Intensity and duration**
Initial or first-line treatment	Polyhexamethylene biguanide (PHMB) 0.06–0.08%	Hourly drops during the day for 5 days, then 8 times a day for 7 days, then 6 times a day for 7 days, then 4 times a day until resolved (often for several months)
If PHMB is not available	Chlorhexidine 0.02% **and** propamidine 0.1%	As above. Monitor for possible side effects including epithelial toxicity and rarer side effects including peripheral ulcerative keratitis. Use minimum frequency possible.
To prevent secondary bacterial infection while there is an epithelial defect	Moxifloxacin, ciprofloxacin, or ofloxacin eye drops.	Administer 4 times a day until the epithelium or ulcer has healed

## Management of complications secondary to microbial keratitis

Despite appropriate treatment, some patients with microbial keratitis develop complications including descemetoceles (exposure or protrusion of Descemet's membrane) and corneal perforations. For some patients, the cornea continues to melt. The recommended treatment for some of these complications would be to do an amniotic membrane transplantation, seal the perforation using tissue glue, use a bandage contact lens, or perform a therapeutic keratoplasty. These are, however, not available in many low-resource settings.

**Figure 1:**
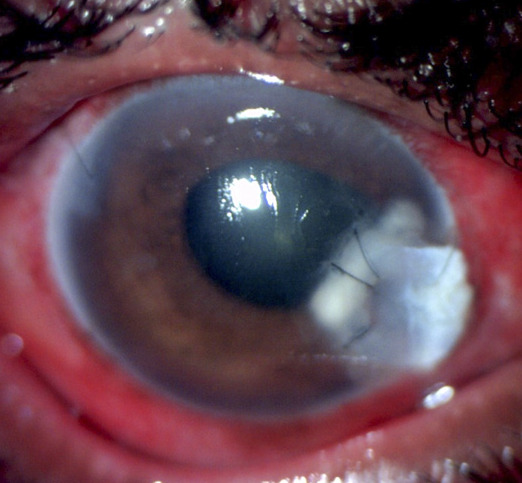
Conjunctival flap to address eccentric corneal ulcer perforation.

**Figure 2:**
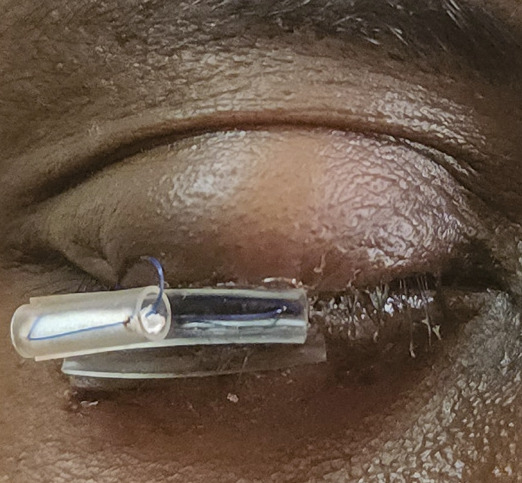
Temporary tarsorrhaphy using the drawstring method makes it possible to examine the eye without the need for repeat surgery.

In resource-limited facilities, small perforations and descemetoceles can be managed using procedures like conjunctival flaps (Figure 1) and temporary tarsorrhaphy, or surgical eyelid closure. The use of conjunctival flaps in patients with active corneal infection is not encouraged as it may aggravate the infection. We recommend that these patients first undergo a temporary tarsorrhaphy and, once there are no signs of infection (negative microscopy), the conjunctival flap is done. The drawstring procedure of temporary tarsorrhaphy (Figure 2) is recommended, because it enables us to loosen the sutures to examine the eye without the need for repeat surgery. See issue #89 for detailed guidance on how to perform drawstring tarsorrhaphy: www.cehjournal.org.articles/584.

© CORNEA TEAM SCEH, NEPAL CC BY-NC-SA 4.0

© ABEL EBONG CC BY-NC-SA 4.0

Perforations can be sealed using **autologous Tenon grafts** for perforations of up to 5 mm or **scleral grafts** (preferably) for peripheral corneal perforations. Tenon grafts use tissue from the patient's own eye to treat corneal perforations. The tissue is harvested from the Tenon's capsule: a dense, elastic, fibrous connective tissue in the eye. Scleral grafts use the scleral rim from a corneal donor and can be stored on the shelf in 100% ethanol. They offer structural support and there is little chance of rejection.

**PART 3 Fungal keratitis: two patients with different outcomes**

**Patient 1: Improvement with chlorhexidine 0.2% eye drops**

An eighteen-year-old female patient presented with a chief complaint of eye pain, redness, and photophobia that had lasted for 15 days. There was no memorable history of ocular trauma. She had received one antibiotic in the form of eye drops for 6 days from the local pharmacy shop; however, no documents were available to confirm which treatment had been given.

**Figure 3:**
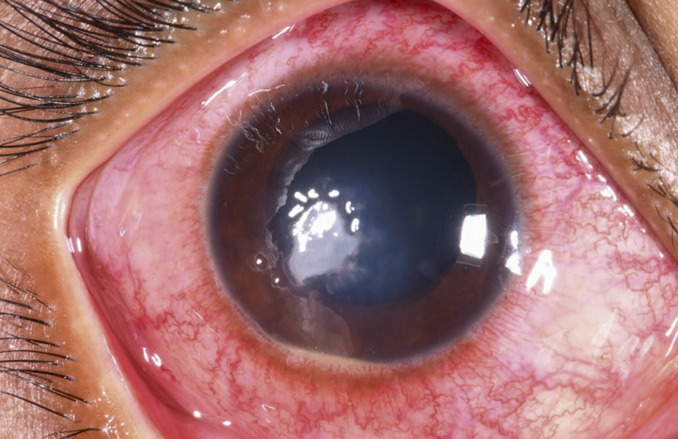
Stromal infiltration with hypopyon at presentation.

On examination, she had paracentral stomal infiltration (2.8 × 2.2 mm in size) involving about 50% of the total stomal thickness, along with a hypopyon of 0.4 mm (Figure 3). On microbiological examination, septate fungal hyphae were seen on KOH mount and culture but the species could not be identified.

© CORNEA TEAM SCEH, NEPAL CC BY-NC-SA 4.0

**Figure 4:**
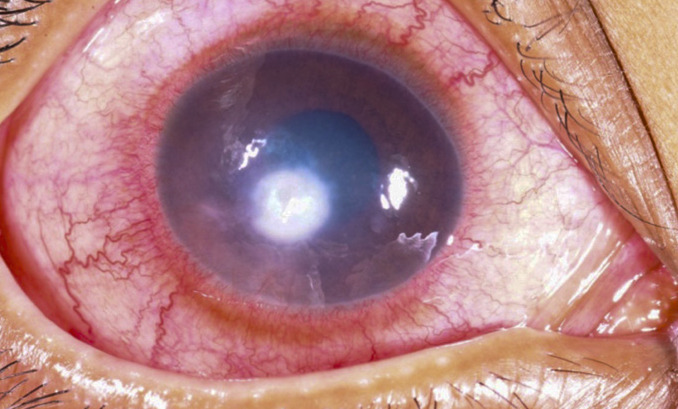
Resolving stromal infiltration on treatment with antifungal (chlorhexidine 0.2% eye drops) at 2-month follow-up.

**Figure 5:**
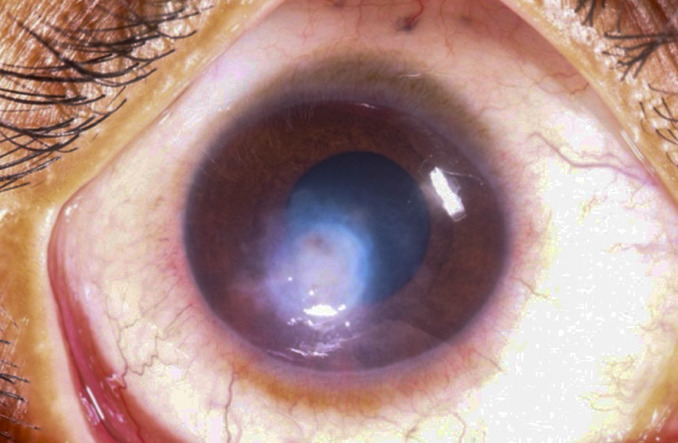
Ongoing scarring at 3-month follow-up.

The patient was prescribed chlorhexidine 0.2% eye drops every hour, day and night, initially, then tapered as per the standard protocol. The patient was admitted to ensure compliance with the frequent drop application and to monitor response to treatment. This also ensures the patient can attend follow up, as they often have to travel considerable distances. The patient showed marked improvement on subsequent follow-up evaluation at 2 months (Figure 4) and 3 months (Figure 5), although there was ongoing scarring at 3 months.

© CORNEA TEAM SCEH, NEPAL CC BY-NC-SA 4.0

© CORNEA TEAM SCEH, NEPAL CC BY-NC-SA 4.0

**Patient 2: Poor outcome despite treatment**

A fifty-two-year-old female patient presented with reduced vision with perception of light in the affected eye. There was history of foreign body sensation, redness, and watering. These symptoms were present 22 days before she presented at the eye hospital. There was a history of the use of some eye drops for 10 days before presentation, although it was unclear which ones.

**Figure 6:**
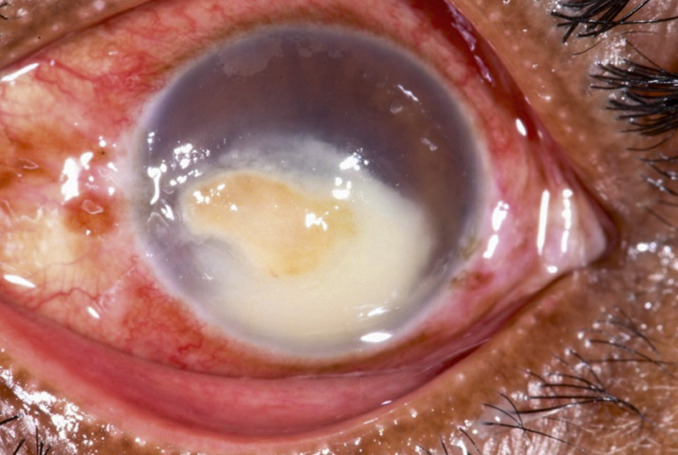
Late presenting subtotal stromal infiltration.

There was extensive corneal stromal involvement (5.5 mm × 7.0 mm) with the presence of an endothelial plaque (Figure 6). Microbiological tests revealed *Aspergillus* infection.

© CORNEA TEAM SCEH, NEPAL CC BY-NC-SA 4.0

**Figure 7:**
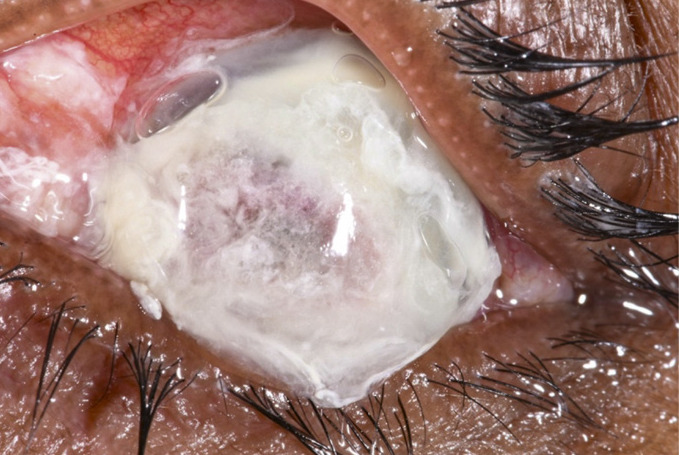
Perforated corneal ulcer.

The patient was admitted and topical natamycin 5% was started along with a systemic antifungal agent (oral voriconazole). Despite these medications, stromal infiltration worsened. Topical voriconazole 1% was added after a week. Unfortunately, 5 days later the affected eye had perforated (Figure 7) and an evisceration was performed.

© CORNEA TEAM SCEH, NEPAL CC BY-NC-SA 4.0


**These two patients highlight how fungal keratitis can lead to distinctly different clinical outcomes. There are several factors that can explain this: the delayed presentation for the second patient, the pathogen or microorganism involved (*Aspergillus* can be more challenging to treat and natamycin may be less effective), and the treatment given. The second patient may also have received topical steroid drops from a local pharmacy, which leads to worse outcomes.**
